# HDX-guided EPR spectroscopy to interrogate membrane protein dynamics

**DOI:** 10.1016/j.xpro.2022.101562

**Published:** 2022-07-18

**Authors:** Benjamin J. Lane, Bolin Wang, Yue Ma, Antonio N. Calabrese, Hassane El Mkami, Christos Pliotas

**Affiliations:** 1 Astbury Centre for Structural Molecular Biology, University of Leeds, Leeds LS2 9JT, UK; 2 School of Biomedical Sciences, Faculty of Biological Sciences, University of Leeds, Leeds LS2 9JT, UK; 3 School of Molecular and Cellular Biology, Faculty of Biological Sciences, University of Leeds, Leeds LS2 9JT, UK; 4 School of Physics and Astronomy, University of St Andrews, St Andrews KY16 9SS, UK

**Keywords:** Biophysics, Cell Membrane, Molecular Biology, Protein Biochemistry, Protein expression and purification, Structural Biology, Mass Spectrometry

## Abstract

Solvent accessibilities of and distances between protein residues measured by pulsed-EPR approaches provide high-resolution information on dynamic protein motions. We describe protocols for the purification and site-directed spin labeling of integral membrane proteins. In our protocol, peptide-level HDX-MS is used as a precursor to guide single-residue resolution ESEEM accessibility measurements and spin labeling strategies for EPR applications. Exploiting the pentameric MscL channel as a model, we discuss the use of cwEPR, DEER/PELDOR, and ESEEM spectroscopies to interrogate membrane protein dynamics.

For complete details on the use and execution of this protocol, please refer to Wang et al. (2022).

## Highlights

• Protocols for an integrated EPR-based approach to study membrane protein dynamics
• Instructions for the sample preparation of spin-labeled membrane proteins
• Used HDX-MS as a precursor to guide spin labeling strategies for EPR methods
• Probed solvent accessibility at the single-residue level by ESEEM

## Abstract (editor highlights)

Publisher’s note: Undertaking any experimental protocol requires adherence to local institutional guidelines for laboratory safety and ethics.

## Graphical abstract

**Figure:**
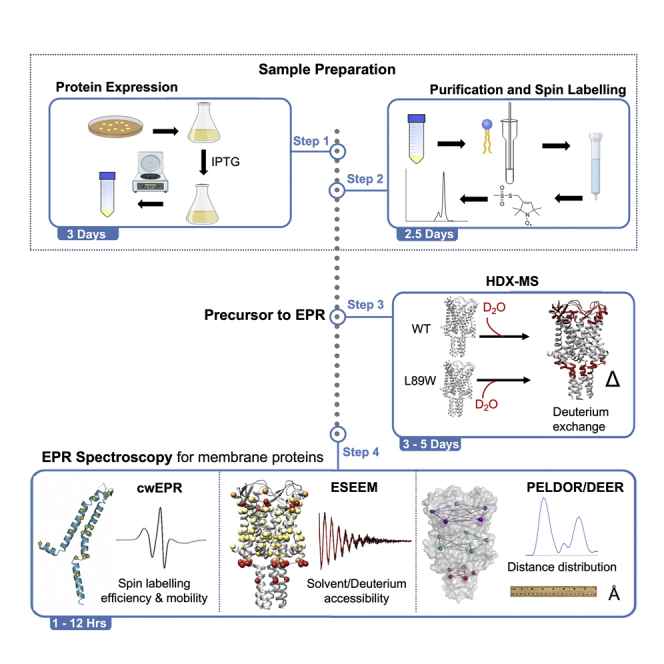

## Before you begin

The protocol below describes the specific steps required for the preparation of integral membrane protein samples using the MscL ion channel as a model system. These steps include the purification and site-directed spin labeling (SDSL) steps required for the EPR spectroscopy experiments. It comprises detailed protocols for assessing spin labeling and label mobility by continuous wave electron paramagnetic resonance (cwEPR) (Ward et al., 2014; Pliotas et al., 2012; Roosild et al., 2010; Branigan et al., 2013; Hubbell et al., 2000; Pliotas, 2017) and the set-up of pulsed-EPR measurements to probe the accessibility of membrane protein residues by electron spin echo envelope modulation (ESEEM) spectroscopy at single-residue resolution under different stimuli, substrates and conditions (Wang et al., 2022; Kapsalis et al., 2020; Liu et al., 2016; Cieslak et al., 2010; Volkov et al., 2009; Pliotas, 2017). The same protein samples could then also be used to perform pulsed electron-electron double resonance (PELDOR) or double electron-electron resonance (DEER) spectroscopy distance measurements to assess the proteins fold, oligomeric state and conformational ensemble in a native environment (Hartley et al., 2021; Michou et al., 2019; Pliotas, 2017; Kapsalis et al., 2019, 2020; Ackermann et al., 2017; Ward et al., 2014; Pliotas et al., 2012; Bountra et al., 2017; Wingler et al., 2019; Timachi et al., 2017; Joseph et al., 2019; Debruycker et al., 2020; Yardeni et al., 2019; Georgieva et al., 2013; Valera et al., 2016; Jeschke, 2012; Schiemann and Prisner, 2007; Martens et al., 2016). Similar sample preparation protocols can be used for peptide resolution Hydrogen Deuterium Exchange mass spectrometry (HDX-MS) measurements, which due to high amino acid sequence coverage, label-free capabilities and relatively fast acquisition times dovetails well with modern EPR structural biology methods and can act as a precursor to guide site-directed spin labeling residue selection (Hartley et al., 2021; Martens et al., 2019; Calabrese et al., 2020; Cornwell et al., 2018; Calvaresi et al., 2021; Martens and Politis, 2020; Wang et al., 2022). Indeed, HDX-MS could highlight global protein regions which experience changes in solvent accessibility when they undergo structural transitions. These sites could in turn constitute ideal candidates for spin labeling to enable the implementation of pulsed EPR methods which provide molecular detail mechanistic insights (Hartley et al., 2021). We here describe solvent accessibility analysis of 26 distinct protein residues, spanning all MscL ion channel domains. This has allowed us to probe conformational changes of the membrane protein, triggered by a single residue modification (L89W). The L89W modification was introduced at the entrance of a pocket that is essential for lipid access, which is crucial to the mechanical sensing and response of the channel to membrane tension. This modification has been shown to stabilize an expanded MscL state by impeding lipid access to the pockets (Wang et al., 2022; Kapsalis et al., 2019, 2020; Pliotas et al., 2015; Pliotas and Naismith, 2016). For different proteins, expression and purification protocols can be tailored by the addition of the steps required to introduce the spin-label to proteins for PELDOR/DEER and/or ESEEM spectroscopy applications. The protocols described here for spin labeling, cwEPR and pulsed EPR (ESEEM and PELDOR/DEER) spectroscopies are not restricted to membrane proteins and could equally be applied to soluble proteins with no modification. These techniques can be used to address a wide variety of questions such as the effect of lipids on protein conformation and/or oligomerization, localization of ligand/substrate binding, allosteric responses, and folding dynamics (Hartley et al., 2021).

### Preparation for the expression of integral membrane proteins (*M tuberculosis* MscL ion channel, TbMscL)

**Timing: 0.5 days**

1. Prepare 10 mL ampicillin stock with a concentration of 100 mg/mL.
2. Use the ampicillin stock to prepare Luria-Bertani (LB) agar plates (1 L contains 10 g tryptone, 5 g yeast extract, 10 g NaCl, and 12 g agar) supplemented with ampicillin (final concentration 100 μg/mL).
3. Make 1 L of LB media in a Duran bottle (1 L contains 10 g tryptone, 5 g yeast extract and 10 g NaCl).
4. Prepare 9 × 500 mL LB media in 2 L growth flasks, cover with foil and autoclave.
5. Use site-directed mutagenesis to introduce required mutations into the pJ411:140126-TbMscL for HDX-MS and ESEEM spectroscopy. Liu et al. provide a recommended protocol for site-directed mutagenesis, and Pliotas et al. provides detailed consideration for the selection of labeling sites (Liu and Naismith, 2008; Hartley et al., 2021; Pliotas, 2017).

***Note:*** We use a TbMscL L89W mutation, which stabilizes a subconducting state, for comparison with WT TbMscL using HDX-MS and solvent accessibility measurements via ESEEM spectroscopy (Wang et al., 2022; Kapsalis et al., 2019, 2020). For ESEEM we introduced multiple other mutations and the primers we used can be found in the key resources table.

6. Prepare BL21(DE3) competent cells.

### Preparation for the purification and spin labeling of MscL

**Timing: 0.5 Days**

7. Prepare the following stock solutions.
  a. Prepare 500 mL of a 3 M NaCl stock, filter using 0.22 μM filter paper and store at 4°C to pre-cool for ice-cold buffers. This can be stored at 4°C for up to 3 months.
  b. Prepare 500 mL of a 500 mM pH 7.5 sodium phosphate stock, filter using 0.22 μM filter paper, and store at 4°C for up to 3 months.
  c. Prepare a stock of 50% glycerol.
  d. Prepare 500 mL of 1× PBS.
  e. Prepare a 50 mL 1 M stock of imidazole.
  f. Prepare a 20 mL stock of 1% DDM. This can be stored at −20°C for up to 6 months.
  g. Prepare 70% EtOH for cleaning of the cell disruptor.
  h. Prepare 1% Teepol for cleaning of the cell disruptor.
  i. Prepare a 100 mM MTSSL stock solution in DMSO and store it at −80°C.
  j. Fill a 1 L Duran bottle with mqH_2_O and store at 4°C for making ice-cold buffers.
8. Ensure the Ni-NTA gravity column is recharged.
  a. Prepare 0.5 M NaOH and use to wash the column with 15× column volumes.
  b. Wash the column using 15× the column volume of mqH_2_O.
  c. Prepare stripping buffer (50 mM sodium phosphate, 300 mM NaCl, 100 mM EDTA, pH 8.0) and wash the column with 10× column volume.
  d. Wash with 20× the column volumes of mqH_2_O.
  e. Prepare 100 mM NiSO_4_ in mqH_2_O and wash the column with 2× the column volume.
  f. Wash the column with 10× the column volume of mqH_2_O.

### Preparation for ESEEM spectroscopy

**Timing: 4–6 h**

***Note:*** The cooling down of the cryostat in the EPR spectrometer takes about 4–6 h. This can vary depending on how the cryostat is cooled down. The cooling procedure is often performed by using either liquid nitrogen or helium and in our case part of the experiments was conducted by using a cryogen-free system. Getting the sample ready for measurements takes about 5–10 min.

9. Ensure you have a clean and dry EPR tube.
10. Ensure the protein sample is spin-labeled.
11. Ensure you have enough liquid nitrogen or helium to conduct your experiment. The sample cryostat should be pre-cooled prior to loading the sample.

### Preparation for HDX-MS

**Timing: 0.5 days**

12. Prepare the quench buffer (50 mM potassium phosphate, 300 mM NaCl, 0.1% DDM pH 2.2, ice-cold). To store aliquot into 10 mL aliquots and store at −20°C for up to 6 months. Thaw immediately before use.
13. Prepare the deuterated labeling buffer (50 mM potassium phosphate, 300 mM NaCl pH 7.4, 0.05% DDM) with D_2_O. To store aliquot into 10 mL aliquots and store at −20°C for up to 6 months. Thaw immediately before use.
14. Prepare the pepsin column wash buffer (5% acetonitrile, 1.2% formic acid, 0.05% DDM).
15. Calibrate the mass spectrometer following the manufacturer’s instructions.
16. Ensure your mass spectrometer is interfaced with a liquid chromatography system setup for HDX, including a switching valve to be able to perform online pepsin digestion followed by peptide trapping, and a cooled compartment so that reverse phase chromatography can be performed at ca. 0°C. Here we have used a Waters LC-MS system with binary and auxiliary solvent managers, HDX manager and LEAP robot, interfaced with a Synapt G2-Si high-resolution mass spectrometer.
17. For any new protein to be analyzed, perform triplicate injections without labeling the protein and check to ensure adequate sequence coverage. See the method below.

## Key resources table

**Table:** 

REAGENT or RESOURCE	SOURCE	IDENTIFIER
**Bacterial and virus strains**		

BL21(DE3) Competent Cells	Thermo Fisher Scientific	Cat# EC0114

**Chemicals, peptides, and recombinant proteins**		

N-Dodecyl-b-D-Maltopyranoside (DDM), anagrade	Anatrace or Glycon	Cat# D310 or D97002
S-(2,2,5,5-tetramethyl-2,5-dihydro-1H-pyrrol-3-yl)methyl methanesulfonothioate (MTSSL)	Santa Cruz or Toronto Research Chemicals	Cat# 81213-52-7 or O875000
Glycerol	Fisher Scientific	G0650
Imidazole BioUltra	Sigma-Aldrich	56749
Sodium Chloride (NaCl)	Sigma-Aldrich	71383
LB Agar, Miller	Sigma-Aldrich	L3027
LB media, Miller	Sigma-Aldrich	L3152
TCEP-HCl	Thermo Scientific	20491
Phosphate buffered saline (PBS)	Sigma-Aldrich	P4417
Sodium Hydroxide (NaOH)	Fisher Scientific	BP359
Sodium phosphate dibasic dodecahydrate	Honeywell	04273
Sodium phosphate, monobasic dihydrate	Fisher Scientific	12645107
IPTG Dioxane Free	Formedium	367-93-1
Ampicillin	Formedium	69-52-3
D_2_O	Cambridge Isotope Laboratories	DLM-4-100
H_2_O (LC-MS Grade)	Supelco	1.15333
Acetonitrile	Supelco	1.00029
Formic acid	Supelco	5.33002

**Critical commercial assays**		

Ni-NTA Agarose Resin	Invitrogen	Cat# R901-15
Superdex 200 Increase 10/300 GL column Superdex 200 16/600 GL column	Cytiva	Cat# 28-9909-44 Ca# 28-9893-35

**Deposited data**		

MscL and MscL L89W HDX mass spectrometry data	Wang et al., 2022	ProteomeXchange PXD021983https://doi.org/10.1016/j.str.2021.12.004
MscL (various) mutants ESEEM data	Wang et al., 2022	http://archive.researchdata.leeds.ac.uk/777/https://doi.org/10.1016/j.str.2021.12.004

**Oligonucleotides**		

Primer: MscL N13C Forward CGCGGTTGTATTGTTGACTTGGCGGT	Wang et al., 2022	https://doi.org/10.1016/j.str.2021.12.004
Primer: MscL N13C Reverse CAACAATACAACCGCGAGCCAGGAATTC	Wang et al., 2022	https://doi.org/10.1016/j.str.2021.12.004
Primer: MscL L17C Forward TGACTGCGCGGTTGCGGTTGTCATTGG	Wang et al., 2022	https://doi.org/10.1016/j.str.2021.12.004
Primer: MscL L17C Reverse CCGCGCAGTCAACAATATTACCGCGAGC	Wang et al., 2022	https://doi.org/10.1016/j.str.2021.12.004
Primer: MscL V21C Forward TTGCGTGTGTCATTGGTACCGCGTTTACCG	Wang et al., 2022	https://doi.org/10.1016/j.str.2021.12.004
Primer: MscL V21C Reverse CAATGACACACGCAACCGCCAAGTCAACAAT	Wang et al., 2022	https://doi.org/10.1016/j.str.2021.12.004
Primer: MscL V71C Forward GATTTGAATTGCCTGCTGAGCGCCGCTATTAAC	Wang et al., 2022	https://doi.org/10.1016/j.str.2021.12.004
Primer: MscL V71C Reverse GCAGGCAATTCAAATCGATGGTCTGACCACC	Wang et al., 2022	https://doi.org/10.1016/j.str.2021.12.004
Primer: MscL S74C Forward CTGCTGTGCGCCGCTATTAACTTC	Wang et al., 2022	https://doi.org/10.1016/j.str.2021.12.004
Primer: MscL S74C Reverse GGCGCACAGCAGGACATTCAAATC	Wang et al., 2022	https://doi.org/10.1016/j.str.2021.12.004
Primers: remaining primers have been reported	Kapsalis et al. (2019)	https://doi.org/10.1038/s41467-019-12591-x

**Recombinant DNA**		

Plasmid: pJ411:140126-TbMscL	Kapsalis et al. (2019)	https://doi.org/10.1038/s41467-019-12591-x

**Software and algorithms**		

PLGS (v3.0.2)	Waters	https://www.waters.com/waters/en_GB/ProteinLynx-Global-SERVER-%28PLGS%29/nav.htm?locale=en_GB&cid=513821
DynamX (v3.0.0)	Waters	https://www.waters.com/waters/library.htm?locale=en_US&lid=134832928
PAVED	Cornwell et al. (2018)	https://doi.org/10.1007/s13361-018-2067-y
Deuteros	Lau et al. (2019)	https://github.com/andymlau/Deuteros_2.0
MATLAB Curve Fitting Toolbox	N/A	https://uk.mathworks.com/products/curvefitting.ht
Xepr Software	Bruker	https://www.bruker.com/en/products-and-solutions/mr/epr-instruments/epr-software.html

**Other**		

Vivaspin-2 (100 kDa MWCO) Concentrator	Sartorius	Cat# VS0241

## Materials and equipment

Key Buffers.

**Table:** Solubilization buffer

Reagent	Final concentration	Amount
Na-phosphate buffer pH 7.5 (0.5 M)	50 mM	10 mL
NaCl (3 M)	300 mM	10 mL
Glycerol (50%)	10%	20 mL
Imidazole (1 M)	50 mM	5 mL
mqH_2_O	n/a	55 mL
DDM detergent	1.5% (w/v)	1.5 g
**Total**		**100 mL**

Should be made fresh each time and cool to 4°C. Buffers containing detergent should not be stored more than a week.

**Table:** 2× purification buffer

Reagent	Final concentration	Amount
Na-phosphate buffer pH 7.5 (0.5 M)	50 mM	4 mL
NaCl (3 M)	300 mM	4 mL
Glycerol (50%)	10%	8 mL
1% DDM Stock	0.1%	2 mL
mqH_2_O	n/a	2 mL
**Total**		**20 mL**

Make fresh each time.

**Table:** Wash buffer (for HDX-MS purification)

Reagent	Final concentration	Amount
2× Purification Buffer	1×	12.5 mL
Imidazole (1 M)	50 mM	1.25 mL
mqH_2_O	n/a	11.25 mL
**Total**		**25 mL**

Make fresh each time and cool to 4°C.

**Table:** Wash buffer (for ESEEM spectroscopy purification)

Reagent	Final concentration	Amount
Na-phosphate buffer pH 7.5 (0.5 M)	50 mM	5 mL
NaCl (3 M)	300 mM	5 mL
Glycerol (50%)	10%	10 mL
1% DDM Stock	0.05%	2.5 mL
mqH_2_O	n/a	27.5 mL
**Total**		**50 mL**

Make fresh each time and cool to 4°C.

**Table:** Elution buffer

Reagent	Final concentration	Amount
2× Purification Buffer	1×	3 mL
Imidazole (1 M)	300 mM	1.8 mL
mqH_2_O	n/a	1.2 mL
**Total**		**6 mL**

Make fresh each time and cool to 4°C.

**Table:** SEC buffer

Reagent	Final concentration	Amount
Na-phosphate buffer pH 7.5 (0.5 M)	50 mM	25 mL
NaCl (3 M)	300 mM	25 mL
mqH_2_O		187.5 mL
1% DDM Stock	0.05% (w/v)	12.5 mL
**Total**		**250 mL**

Should be made fresh each time and cooled to 4°C. The SEC buffer should be made up to 237.5 mL with mqH_2_O and degassed before the addition of the DDM detergent. This should not be stored more than week.

**Table:** HDX quench buffer

Reagent	Final concentration	Amount
K_2_HPO_4_	50 mM	436 mg
KH_2_PO_4_		340 mg
NaCl (3 M)	300 mM	10 mL
10% (w/v) DDM	0.05% (w/v)	0.5 mL
Adjust pH to 1.8 (with HCl)		
Water		Make up to 100 mL
**Total**		**100 mL**

To store aliquot into 10 mL aliquots and store at −20°C for up to 6 months. Thaw immediately before use.

**Table:** HDX buffer

Reagent	Final concentration	Amount
K_2_HPO_4_	50 mM	436 mg
KH_2_PO_4_		340 mg
NaCl (3 M) (in H_2_O or D_2_O)	300 mM	10 mL
10% (w/v) DDM (in H_2_O or D_2_O)	0.05% (w/v)	0.5 mL
Adjust pH/pD to 7.4 (with HCl/NaOH or DCl/NaOD)		
Water or D_2_O		Make up to 100 mL
**Total**		**100 mL**

To store aliquot into 10 mL aliquots and store at −20°C for up to 6 months. Thaw immediately before use.

## Step-by-step method details

### Expression of MscL in BL21(DE3) *E.coli* cells

**Timing: 3 days**

This protocol follows the transformation of the TbMscL plasmid into BL21(DE3) and the expression of the protein. The plasmid could be either for the expression of WT or mutant TbMscL.

1. Transform the pJ411:140126-TbMscL plasmid into BL21(DE3) cells.
  a. Remove BL21(DE3) competent cells (50 μL) in an Eppendorf from the freezer and thaw.
  b. Add 1 μL of the expression plasmid (∼100 μg/mL) and incubate on ice for 20 min, tapping the tube occasionally to mix.
  c. Heat shock the E. *coli* cells by placing the tube in a water bath or hot plate at 42°C for 1 min.
  d. Place the tube back on ice for 2 min.
  e. Add 350 μL of LB media (without antibiotic) to the bacteria and grow, shaking at 37°C for 1–2 h.
  f. Plate 100 μL onto a 10 cm LB agar plate containing the relevant antibiotic (ampicillin at 100 μg/mL for the pJ411:140126-TbMscL plasmid).
  g. Incubate the plates 12–20 h at 37°C.
2. Prepare 2 × 10 mL precultures from a single colony, by using sterile pipette tips to inoculate 10 mL supplemented with ampicillin (100 μg/mL) in 50 mL falcon tubes.
3. Grow the precultures 12–20 h at 37°C and 180 rpm in a shaker.
4. Add 18 mL of the overnight preculture to 500 mL of LB in a 2 L flask to generate a secondary preculture.
5. Grow the secondary preculture at 37°C in a shaking incubator (180 rpm) until the OD_600_ reaches 0.6–0.8 (approximately 1.5 h).
6. Transfer 50 mL of the secondary preculture to each of the eight remaining 2 L flasks containing 500 mL LB.
7. Grow the cultures (37°C, 180 rpm) until the OD_600_ reaches 0.6–0.8, then cool the flasks to 25°C and induced with 1 mM IPTG.
8. Express the protein for 3.5 h by incubating at 25°C, 180 rpm.
9. Harvest the cells by centrifugation at 4,000 × *g* (Sorval flasks, JLA 8.1 rotor) and store at −80°C.

### Purification and spin labeling of the MscL protein

**Timing: 2–2.5 days**

This protocol explains the purification of the integral MscL membrane protein and preparation for downstream analysis via EPR spectroscopy and HDX mass spectrometry. The steps (step 30) for the spin-labeling of the membrane (or soluble) protein are key for its analysis in EPR spectroscopy. Specific purification protocols for alternative proteins can be followed with the addition of these spin-labeling steps in order to study the protein using EPR spectroscopy. In this protocol, the MscL protein is solubilized in DDM detergent, but the protein could also be reconstituted in model membrane systems such as nanodiscs for downstream analysis using HDX-MS and EPR spectroscopy. We have previously studied MscL in nanodiscs and liposomes using both ESEEM and PELDOR spectroscopy (Kapsalis et al., 2019, 2020). For HDX-MS in lipid bilayer mimics it may be necessary to include an additional lipid removal step after deuterium labeling and before MS analysis (Martens et al., 2019).

10. Thaw the pellet and resuspend in PBS (20 mL per 500 mL culture) by vortexing.
11. Transfer to a Duran bottle, add a magnetic stirrer bar and leave stirring in the cold room for 15–20 min to homogenize the cell suspension.
12. Use a cell disruptor to lyse the bacterial cells at 4°C. We use an Avestin Emulisflex C3 continuous flow cell disruptor pulsing at 15,000 psi and pass through twice.
13. Once the cells have been lysed, clean the disruptor by passing through mqH_2_O, followed by 1% Teepol, followed by mqH_2_O.
14. Transfer the lysed cells to 50 mL falcons and centrifuge at 4,700 × *g*, 4°C for 20 min in a benchtop centrifuge.

***Note:*** Place enough ultracentrifuge tubes for the volume of cell lysis on ice to cool during the 20 min centrifuge step.

15. Transfer the supernatant to pre-weighed Beckman ultracentrifuge tubes. Balance the tubes and ultracentrifuge at 100,000 × *g*, 4°C for 1 h.
16. Discard the supernatant, weigh the tubes with the pellet, and calculate the mass of the membrane.

***Note:*** Place a glass homogenizer and spatula (upside down) on ice to chill during the ultracentrifuge step.

17. Prepare 25 mL of solubilization buffer per 1 g of membrane (50 mM Na-phosphate buffer pH 7.5, 300 mM NaCl, 10% glycerol, 50 mM imidazole, 1.5% DDM detergent) and place on ice. This should be made fresh each time. Troubleshooting 1.
18. Divide half of the solubilization buffer between the ultracentrifuge tubes, later using an equal amount to wash each of the tubes and remove the remaining membrane.
19. Use a spatula to slowly scrape the membrane off the side of the tube and decant into the pre-cooled glass homogenizer.
20. Homogenize the membranes until there are no visible pieces of membrane.
21. Transfer to 50 mL falcon tubes, close the lids tightly, then place on a roller at 4°C for 1 h.
22. Centrifuge the solubilized membrane at 4,700 × *g*, 4°C for 20 min in a benchtop centrifuge.
23. Add the solubilization buffer to a gravity column containing 1 mL (2 mL suspension) of pre-equilibrated Ni^2+^-NTA resin and allow it to pass through.
24. Prepare gel filtration (SEC) buffer (50 mM Na-phosphate buffer pH 7.5, 300 mM NaCl, 0.05% DDM) and degas for at least 1 h. We make ∼250 mL for our Cytiva Superdex 200 10/300 columns. This should be prepared fresh each time.
25. Once degassed, move to 4°C to equilibrate the temperature.
26. If purifying for HDX-MS, prepare 20 mL of the 2× purification buffer (100 mM Na-phosphate buffer pH 7.5, 600 mM NaCl, 20% glycerol, 0.1% DDM). This should be prepared fresh each time.
  a. Then use the 2× purification buffer to make the wash buffer (50 mM Na-phosphate buffer pH 7.5, 300 mM NaCl, 10% glycerol, 0.05% DDM, 50 mM imidazole) and elution buffer (50 mM Na-phosphate buffer pH 7.5, 300 mM NaCl, 10% glycerol, 0.05% DDM, 300 mM imidazole):
    i. Prepare 25 mL of wash buffer by taking 12.5 mL of the 2× purification buffer, adding 1.25 mL of the 1 M imidazole, and topping up with 11.25 mL of mqH_2_O.
    ii. Prepare 6 mL of elution buffer by taking 3 mL of the 2× purification buffer, adding 1.8 mL of the 1 M imidazole stock, and topping up with 1.2 mL of mqH_2_O.
27. When purifying for EPR spectroscopy, a 2× purification buffer is not needed as more degassed wash buffer is needed for spin labeling. Prepare 50 mL of wash buffer (50 mM Na-phosphate buffer pH 7.5, 300 mM NaCl, 10% glycerol, 0.05% DDM, 50 mM imidazole) and degas for at least 1 h.
  a. Use the degassed wash buffer and the previously prepared 100 mM MTSSL stock to make 4 mL of MTSSL wash buffer (50 mM Na-phosphate buffer pH 7.5, 300 mM NaCl, 10% glycerol, 0.05% DDM, 50 mM imidazole, 1.5 mM MTSSL).
  b. Use the degassed wash buffer and the previously prepared 100 mM TCEP stock to make 4 mL of the TCEP wash buffer (50 mM Na-phosphate buffer pH 7.5, 300 mM NaCl, 10% glycerol, 0.05% DDM, 50 mM imidazole, 3 mM TCEP).
28. If purifying for EPR spectroscopy and not using 2× purification buffer, prepare 6 mL of elution buffer (50 mM Na-phosphate buffer pH 7.5, 300 mM NaCl, 10% glycerol, 0.05% DDM, 300 mM imidazole).
29. Once the solubilization buffer containing the protein has passed through the column, pass through wash buffer. If preparing protein for HDX-MS pass through 25 mL of wash buffer, then skip step 30 and leave the column in wash buffer until you can continue. If preparing protein for EPR (cwEPR, PELDOR/DEER and ESEEM) spectroscopy, initially pass through 10 mL of the wash buffer then continue with the labeling steps.

**CRITICAL:** Ensure the nickel bed is covered with buffer at all times.

***Optional:*** Equilibrate the SEC column with 1.2 column volumes (CV) of SEC buffer before pausing. If not, it can be equilibrated during the elution steps the following morning.

30. The following labeling steps are only for protein that will be analyzed using EPR (including cwEPR, PELDOR/DEER and ESEEM) spectroscopy experiments:
  a. After the initial wash, pass 4 mL of the TCEP buffer through the column.
  b. When the TCEP wash buffer has almost passed through, add 5 mL of wash buffer to wash away most of the TCEP.
  c. Then add 4 mL of the MTSSL wash buffer and pass-through half its volume to ensure it replaces the buffer on the nickel bed (∼2 mL) before capping the column.
  d. Remove the Ni^2+^-NTA column from 4°C and allow it to sit at 16°C–25°C for 15 min.
  e. Return the column to 4°C and leave for 12–20 h, ensuring the nickel bed is completely covered with buffer.
  f. The next morning, uncap the column and allow the next ∼2 mL of the MTSSL buffer to replace the buffer on the nickel bed.
  g. Cap the column and move to 16°C–25°C for 15 min.
  h. Then wash with 20 mL of wash buffer to remove any excess MTSSL label.

***Note:*** Care must be taken to slowly add the different wash buffers by pipetting down the side of the column. This prevents disturbing the nickel bed and mixing of the different wash buffers.

**Pause point:** The Ni^2+^-NTA column containing the bound protein can be left in wash buffer for 12–20 h until the following morning.

31. Start the equilibration of the SEC column with 1.2 CV of SEC buffer if it was not previously equilibrated.
32. Following the wash steps, carefully add the elution buffer to the column without disturbing the nickel bed. Elute 8 × 1 mL fraction of purified protein (E1-E8).
33. Measure the UV/Vis spectra for E1-E8 on a nanodrop for an estimate of protein concentration in each of the fractions.
34. Combine fractions containing protein and use a Vivaspin-2 concentration with a 100 KDa MWCO to concentrate the protein to a final volume of ∼800 μL at a speed of 3,500 × *g* at 4°C.
35. Inject the concentrated sample onto the Superdex 200.
36. Increase 10/300 GL column pre-equilibrated with 1.2 CV SEC buffer and run at 0.5 mL/min. MscL elutes at 12 mL on this column or 65 mL on a 16/60 column.
37. Pool the MscL protein containing fractions and concentrate the protein ∼50–100 μM monomer concentration for EPR spectroscopy or 16 μM for HDX-MS.

### HDX-MS

**Timing: 3–5 days**

HDX is a label-free methodology for the analysis of solvent accessibility and dynamics (Englander and Kallenbach, 1983). Detection methods could vary from NMR spectroscopy which provides atomic resolution information in favorable cases (Cavagnero et al., 2000; Cawood et al., 2022) or mass spectrometry-based approaches that typically offer peptide-level resolution. When a protein is incubated with a deuterated solvent, labile hydrogens are exchanged with deuterium. The rate of this exchange is dependent on conditions such as pH, temperature, hydrogen bonding and on protein conformation. For large protein complexes such as integral membrane proteins, MS-based methods are preferred to NMR (Martens et al., 2019; Calvaresi et al., 2021; Wang et al., 2022; Calabrese et al., 2020). In this approach the uptake of deuterium takes place in solution, followed by enzymatic digestion and the increase in mass is measured for each digested peptide. Typically, differential HDX-MS is performed where this extent of deuterium uptake is measured in a minimum of two conditions (e.g., two different protein variants, with/without added ligand). Changes in deuterium uptake between these two conditions can then be determined. This information can then be mapped onto the protein structure to inform on changes in protein dynamics. We used this technique to explore the structural transitions that occur between WT MscL (closed) and an expanded state of MscL stabilized by the mutation L89W (Wang et al., 2022; Kapsalis et al., 2019). Dynamic regions of the protein highlighted by peptide level HDX-MS can then be probed in molecular detail and at single-residue resolution of deuterium accessibility using ESEEM (but also PELDOR/DEER for distance measurements) spectroscopic methods. This protocol discusses the process we used to study MscL *via* HDX-MS but the protocol can be used for comparing different states of other proteins and in the presence or absence of substrates (both soluble and membrane).

38. Ensure you have purified protein that is not spin-labeled at a concentration of 16–40 μM.

***Note:*** For MscL, our initial sample (100 μL) was at 16 μM in SEC buffer (50 mM Na-phosphate pH 7.5, 300 mM NaCl, 0.05% DDM). For our HDX-MS experiments of MscL, the protein was solubilized in DDM detergent. While not all solubilizing agents are compatible with HDX-MS, other solubilization methods can be used. Detergents and solubilizing agents other than DDM should be tested, and Triton-X-100 and PEG-based detergents should not be used to avoid PEG contamination of instrumentation.

39. Use an automated liquid handling robot fitted with a gas tight syringe that is coupled to the LC-MS system to perform the following dilutions. Place 5 μL of the MscL sample in a total recovery vial and use the syringe to mix with 95 μL of SEC buffer (50 mM Na-phosphate buffer pH 7.5, 300 mM NaCl, 0.05% DDM).
40. Use the syringe to take 50 μL of the diluted MscL sample and add to 100 μL of quench buffer (50 mM potassium phosphate, 300 mM NaCl, 0.1% DDM pH 2.2, ice-cold) and mix. Immediately inject 50 μL of the sample onto HDX Manager and start the method.

***Note:*** Use the LEAP HDX manager software to program the instrument to perform these dilutions.

***Note:*** During the LC-HDXMS method the sample is passed through (115 μL min^-1^, 20°C) the immobilized pepsin and aspegillopepsin columns connected in series, and the resultant peptides are trapped on a VanGaurd Pre-column Acquity UPLC BEH C18 for 3 min in 0.3% formic acid. The peptides are then passed onto a C18 column (75 μm × 150 mm, Waters Ltd., Wilmslow, Manchester, UK) and separated by gradient elution of 0%–40% MeCN (0.1% v/v formic acid) in H_2_O (0.3% v/v formic acid) over 7 min at 40 μL.min^-1^. Gradient elution and trapping is performed at 0°C to decrease back exchange. The eluate from the HDX-LC system is infused into the electrospray ion source of a Synapt G2Si mass spectrometer. HDMS^E^ and dynamic range extension modes are used to separate the peptides before CID fragmentation in the transfer cell. Typical instrument parameters include capillary voltage 3 kV, cone voltage 40 V, acquisition range 50–2000 m/z, scan time 0.6 s, transfer collision energy (lookup table), lockmass scan interval 30 s, source temperature 100 C, desolvation temperature 250 C, cone gas 50 L/h, desolvation gas 600 L/h, Nebuliser gas 6.5 Bar.

41. While the gradient elution is being performed, wash the pepsin column with wash buffer (3× infections of 50 μL.
42. Repeat steps 39–41 at least two more times to generate at least three technical replicates.
43. Place 5 μL of the MscL sample in a total recovery vial and use the syringe to mix with 95 μL of deuterated labeling buffer (50 mM potassium phosphate, 300 mM NaCl pH 7.4, 0.05% DDM) and incubate at 4°C for a defined incubation period (30 s, 1 min, 2 min, 10 min, 1 h).
44. Use the syringe to take 50 μL of the diluted sample and add to 100 μL of quench buffer (50 mM potassium phosphate, 300 mM NaCl, 0.1% DDM pH 2.2, ice-cold) and mix. Immediately inject 50 μL of the sample onto HDX Manager and start the method. Troubleshooting 3.

***Note:*** Use the LEAP HDX manager software to program the instrument to perform these dilutions.

45. Repeat this process for all desired deuterium incubation periods and repeat each incubation period a minimum of 3 times (3 replicates). For MscL, we used 30 s, 1 min, 2 min, 10 min, and 1 h time periods. The duration of the timescales should be chosen to inform on dynamics at different timescales of motions.
46. Search the data using PLGS (v3.0.2) to generate a list of candidate detected peptides. Import a databank containing the protein sequence as well as the sequence(s) of immobilized proteases used.
47. Create workflow parameter file, selecting the appropriate sequence databank, peptide/fragment tolerance set to automatic, min fragment ion matches per peptide set to 3, minimum fragment ion matches per protein set to 7, and min peptide matches per protein set to 1, and ensure the primary reagent is set to pepsin.
48. Create a processing parameter file set to the appropriate lock for charge 2 (here we used GluFib 785.8426 Da/e) and elution start and end times.
49. Search the obtained data files using these workflow and processing parameters.

**CRITICAL:** Ensure that ion counting output is selected in PLGS under the automation setup settings. This will generate files that can be used in DynamX.

50. Use DynamX (v3.0.0) to determine the differential uptake for each peptide. We used the following filtering parameters for assembling the library: minimum intensity 1000, minimum products per MS/MS spectrum 5, minimum products per amino acid 0.3, maximum sequence length 25, maximum ppm error 5, file threshold 3/3.
51. Manually curate the data. Inspect each mass spectrum for each replicate and ensure the correct isotopic envelope is correctly assigned in full. This will automatically generate a deuterium uptake plot that displays the extent of deuterium uptake vs time.
52. Export the cluster data files from DynamX and import the files into Deuteros (Lau et al., 2019). Use the software to identify peptides with statistically significant increases/decreases in deuterium uptake. We applied 99% and 95% confidence intervals.
53. Prepare wood plots using Deuteros to show either the difference in deuterium uptake at each point, and/or the sum of ΔHDX over all time incubation periods. Filter the peptides to identify those that display a significant uptake difference between the two conditions (WT and L89W).
54. Use Deuteros to generate a script to color a protein structure based on the information (significantly different peptides, not significantly different peptides, areas of no sequence coverage).
55. Import a structure of the protein of interest into PyMol and use the script to color the structure.
56. Plotting the difference data on the protein of interest (in our case MscL) allowed us to visually identify regions of the protein with differences in structural dynamics.

### ESEEM spectroscopy

**Timing: 0.5–8 h**

***Note:*** The ESEEM experiments have been conducted using the X-band frequency on an X and Q-band Bruker ELEXSYS E580 spectrometer with a 4 mm dielectric resonator (MD4). At this frequency, the ESEEM effect is much more pronounced and therefore it is ideal for solvent accessibility studies. For PELDOR/DEER distance measurements Q-band is preferred, as it presents much higher sensitivity resulting in a higher signal to noise ratio and thus shorter data acquisitions. Therefore, in our previous PELDOR/DEER distance measurement studies on MscL, we used a Q-band EPR spectrometer. In principle, the ESEEM experiment itself is much quicker than PELDOR/DEER. However, in some cases to get a good signal to noise ratio (SNR) more averaging time is needed. Usually, poor SNR is often related either to the low spin labeling efficiency or to the fact that the mutant is less exposed to the solvent. Overall, and in our case, the ESEEM experiment running time ranges from 30 min up to 8 h.

ESEEM spectroscopy is a very powerful and robust pulsed-EPR technique that has been widely used to investigate D_2_O permeation profiles in membranes and solvent (or D_2_O) accessibility of proteins (Deligiannakis and Rutherford, 2001; Volkov et al., 2009; Cieslak et al., 2010; Liu et al., 2016; Hartley et al., 2021; Wang et al., 2022; Pliotas, 2017). The technique probes weak hyperfine interactions in a paramagnetic system and has been powerful in the study of metal centers and catalytic mechanisms of metalloproteins. In previous work, we have used the technique to probe membrane protein structure and dynamics by measuring the accessibility of spin-labeled residues to solvent and lipids (Hartley et al., 2021; Wang et al., 2022; Kapsalis et al., 2019, 2020). Following the identification of dynamic regions of MscL using HDX-MS, we used ESEEM spectroscopy to probe these areas further at the single-residue level. This protocol explains the process of checking the labeling efficiency by using cwEPR, and the setting up of ESEEM experiments. The same spin-labeled sample, since it has multiple labels, can be further investigated by other pulsed techniques such as PELDOR (or DEER) spectroscopy with some additional considerations in the setup. PELDOR allows the measurement of distances between spin-labels on a protein, and it provides a robust approach for studying structural changes and dynamics in proteins (Kapsalis et al., 2019, 2020; Pliotas, 2017; Ackermann et al., 2017; Valera et al., 2016; Ward et al., 2014; Pliotas et al., 2012; Bountra et al., 2017; Joseph et al., 2019; Georgieva et al., 2013; Timachi et al., 2017; Wingler et al., 2019; Martens et al., 2016). Distances of between ∼15 and ∼160 Å can be measured using this technique (Ward et al., 2010; Schmidt et al., 2016). However, quality PELDOR distance measurements require a high degree of labeling efficiency, which is not that crucial for obtaining high quality ESEEM data (Wang et al., 2022; Hartley et al., 2021).

57. Load ∼40 μL of the spin-labeled protein sample into a bottom sealed glass EPR tube, using a Hamilton syringe connected to a thin plastic capillary tubing. CW-EPR will be used to check for the labeling efficiency of the protein.
58. Open the ESRStudio software first and then switch on the Bruker Magnettech ESR5000 X-band cwEPR spectrometer at the back.
59. Drag the warm-up recipe to the recipe section under the main control tab (Table 1).

**Table 1:** Warm-up recipe parameters for the Magnettech ESR5000 X-band cwEPR spectrometer

Series type	Single measurement
Magnetic Field (B)	400–450 mT
Sweep Time	600 s
Modulation	0.7 mT
Frequency	100 kHz
Accumulations	1
Microwave Power	50 mW 3.0 dB
60. Click Initialize to connect the spectrometer to the computers. When asked for routine warm-up, click ‘Yes’.
61. After 15 min warm-up, switch on the BioTemperature controller and connected gas nitrogen cylinder and click Initialize again. Click ‘No’ when asked for the routine warm-up.
62. Drag the measuring recipe to the recipe section under Main Control and click Start (Table 2). The machine will start cooling down to 4°C, this should take approximately 30 min.

**Table 2:** Measurement recipe parameters for the Magnettech ESR5000 X-band cwEPR spectrometer

Series type	Temperature series
Magnetic Field (B)	330–345 mT
Sweep Time	60 s
Modulation	0.1 mT
Frequency	100 kHz
Accumulations	40
Microwave Power	10 mW 10.0 dB
Temperature	4°C
63. Once the temperature is stable at 4°C, transfer the EPR containing the protein sample into the holder of the spectrometer. The distance between the center of the sample position and the bottom of the holder should be 6.2 cm.
64. Click Start to measure the sample. The signal intensity should be 25+ on the Bruker Magnettech ESR5000 X-band cwEPR spectrometer. Troubleshooting 2.
65. Once the measurement has finished, take the sample out, add 25 μL deuterated ethylene glycol to the ∼40 μL sample and carefully mix well. Transfer the 65 μL back to the EPR tube.
66. Snap freeze the protein sample in liquid nitrogen to prepare the sample for pulsed EPR techniques.

***Note:*** The pulsed-EPR spectrometer used in this study is a BRUKER E580 ELEXSYS spectrometer and therefore, all the parameters defined in this protocol might not be named in similar ways in other equivalent spectrometers.

67. For the ESEEM experiment, the EPR tube containing the sample is flash-frozen in liquid nitrogen prior to being loaded into a precooled sample cryostat in the EPR spectrometer.
68. Turn on the heat exchanger, the console power, the power supply of the magnet, and the TWT.
69. Start the Xepr software and then go to ‘Acquisition’ and click ‘Connect to spectrometer’.
70. Turning on the resonator:
  a. Open the Microwave Bridge tuning dialog box and switch to tune mode.
  b. Set the microwave attenuator to 30 dB and scan for the cavity dip. The resonator dip is the resonance frequency of the empty cavity.
71. Insert the tube through the small sample holder and screw the latter up to the long rod. Stop the flow cryostat by switching off the diaphragm pump and let it return to ambient pressure using helium gas. Keep flowing the helium gas in the cryostat to prevent icing issues while inserting the sample rod assembly into it.
72. Once the sample is fully inserted in the cavity, resume the cryogen flow by switching on the diaphragm pump. The ESEEM experiments are conducted at 55K/77 K as this range of temperatures constitutes the optimum conditions for the nitroxide.

***Note:*** Although going further down in temperature increases the sensitivity, this will also increase considerably the running time of the experiment. Therefore, a trade-off between sensitivity and the running time of the experiment needs to be considered.

***Note:*** Careful handling is required when inserting the sample into the cavity at low temperatures in order to prevent ice formation inside the cavity.

73. The insertion of the sample into the resonator will shift the cavity dip from the center. Follow the cavity dip by using the frequency slider to bring it back to the center.
74. A pulsed experiment requires an overcoupled resonator to allow a larger excitation bandwidth, and for this move fully up the coupling adjustment arm, which will broaden the dip, and use the frequency slider to keep the resonator dip centered.
75. Once the cavity is overcoupled switch the spectrometer to ‘Pulse mode’ in the ‘Bridge Configuration’ tab on the ‘FT Bridge’ panel.
76. Switch to operate mode in the Microwave Bridge Tuning panel.
77. Make sure that the MW attenuation is set to 60 dB in the receiver Unit tab of the FT EPR Bridge window.
78. Click ‘New Experiment’ in the monitoring panel.
79. Click the Pulse tab, select the Advanced button, and hit the Create button.
80. Click the activate button to ensure that the parameter changes are immediately actualized.

***Note:*** You have the option to run pulse experiments from tables or from the PulseSPEL (Pulse SPEctroscopy Language) program depending on the type of experiment. Usually, tables are used for basic 1D experiments that do not require phase cycling. PulseSPEL is better for more sophisticated pulse sequences that require phase cycling to remove receiver offsets and unwanted echoes. We use the PulseSPEL program for our experiments as 3-Pulse ESEEM requires a four-step phase cycling. Each type of pulse experiment is associated with a specific PulseSPEL program. Therefore, one should select the associated program for the experiment they aim to use.

**CRITICAL:** Before starting any pulse experiments, as we are using high power MW pulses, a safety procedure is necessary to follow before setting any pulse experiment to avoid damaging the MW amplifier or other electronic components.

81. Run an echo-detected field swept EPR experiment is prior to doing any further pulsed measurements. This aims to acquire an absorption EPR spectrum and its associated first derivative is analogous to the CW EPR spectrum. It is performed by integrating the area under the echo while sweeping the magnetic field. This uses a two-pulse sequence echo:
  p0−d1−p1−d1−echo.
  a. Set the magnetic field around 3460G (X-band) for the nitroxide spin-label.
  b. Set up two pulses echo by programming 16 ns and 32 ns (corresponding to p0 and p1 in PulseSPEL program) at 380 ns (d1) apart.
  c. Adjust both the microwave power and the Shot Repetition Time (SRT) to get a maximum echo.
    ***Note:*** The SRT is the time for allowing the magnetization to go back to the equilibrium between individual experiments and is temperature dependent. The optimum SRT for nitroxide is usually set between 3 and 4 ms.
  d. Adjust the signal phase until most of the echo is in the real channel and the imaginary signal is almost flat.
  e. Activate the run PulseSPEL button in the acquisition panel. Note that the frequency is around 9.8 GHz for X-band.
  f. Load the PulseSPEL program and its corresponding variables definitions (Table 3).

**Table 3:** Variable values and definitions for the setup of the Field Sweep experiment

Variable	Value	Definition
p0	16 ns	Pi/2 pulse length.
p1	32 ns	Pi pulse length.
d1	380 ns	delay.
d0	360 ns	Acquisition trigger
Pg	160 ns	Integration gate
SRT	3000 × 1.02 μs	Shot Repetition time.
H	50	Shot Per Point.
N	1	Number of scans.
  g. Once loaded, compile both programs.
  h. Set the parameters p0, p1, SRT to the values used to optimize the echo and compile the variables definitions program.
  i. Select “2P ESE Field Sweep” experiment from drop-down menu in acquisition tab in the FT EPR parameters window with no phase cycling.
  j. Set the acquisition trigger by adjusting d_0_ in the PulseSPEL program (d_0_ = 360 ns in our case). This is adjusted so that the acquisition should start just before the echo.
  k. Set the length of the acquisition trigger pg to cover the whole echo (pg = 160 ns in our case).
  l. Set the sweep width to 120.00G in the Field tab of the FT EPR parameters window and the number of data points (the number of data point is set in the dimension of the associated experiment in the PulseSPEL program).
  m. Press the Run button and save the spectrum.
82. Next perform the T_m_ relaxation time experiment. This has a similar setup as 2pESEEM and is usually used to get, in addition to the transverse relaxation time, some indication about the ESEEM effects prior to running a 3pESEEM. Troubleshooting 4. The corresponding pulse sequence is:
  p0−d1−p1−d1−echo.

In which d1 is incremented by a defined step called d30 (Table 4).

83. Complete this experiment at a fixed magnetic field position, which in our case, is set at the maximum of the field swept spectrum and monitor the echo intensity by incrementing the position of the second pulse. The PulseSPEL program that was used for the field sweep echo also includes the T_m_ experiment.
84. In FT parameters window and under Field tab adjust the Center Field Value to the value of the field corresponding to the maximum point in the Field swept EPR spectrum.
  a. Change the experiment in the experiment box to 2P_ESE_decay.
  b. Since we are monitoring echo height, we only need to integrate the area around its maximum, which corresponds to the length of the long pulse used in the pulse sequence. Set the integrator gate length to pg = 32 ns (our pi pulse length).
  c. Set the delay between the two pulses d_1_= 200 ns. This is limited by the deadtime of the spectrometer, otherwise it is recommended to use the shortest delay possible.
  d. Set the acquisition trigger position d_0_= 418 ns or 420 ns to cover the 32 ns area symmetric around the highest point of the echo.
    ***Note:*** To save all the optimized variable values in PulseSPEL, you need to click the run button after each modification (Table 4).
    **Table 4:** Variable values and definitions for the setup of the T_m_ experiment

Variable	Value	Definition
p0	16 ns	Pi/2 pulse length.
P1	32 ns	Pi pulse length.
D1	200 ns	delay.
D0	418 ns	Acquisition trigger.
Pg	32 ns	Integration gate.
D30	8 ns	Displacement.
SRT	3000 × 1.02 μs	Shot Repetition time.
H	50	Shot Per Point.
N	1	Number of scans.
  e. Run a quick experiment by clicking the Start button next to the pulse tables in the Patterns panel and watch the echo modulating in the SpecJet panel to check whether the signal is clipped or not. In the case of clipping signal, the video gain needs to be reduced until you are getting a non-clipped echo.
  f. Select 2-step phase cycling. This 2-step phase cycling is needed to suppress any receiver offsets.
  g. Click run and save the data.
  h. Estimate the Tm using fitting functions to datasets in the Xepr program. To do this, load the data and phase it in such a way as to get a flat imaginary signal. Select the real part of the signal and in the Processing menu, click the Fitting submenu, then in the exponentials window choose Exponential Decay. Click the Fit button and the program will fit an exponential curve to the real data.
85. Now we can run the 3-pulse ESEEM experiment.
  ***Note:*** The magnetic field position is set to the highest point of the field swept EPR spectrum for higher sensitivity. In a similar way, once the magnetic field is set, adjust the phase and optimize the echo’s intensity by adjusting both the power and the video gain. When using more than two pulses, more echoes are generated as a result of many combinations between the applied pulses. In the case of 3pESEEM, where 3 pi/2 pulses are applied, five echoes are generated. However, only four are observed after the last pulse. The targeted echo which underpins the hyperfine information is the first echo occurring after the last pulse, known as the stimulated echo. Phase cycling is therefore required to select the desired echo and to suppress unwanted echoes. Both 2pESEEM and 3pESEEM experiments can be performed using their associated PulseSPEL program.
  a. Since 3pESEEM is another experiment, we do need to load its associated PulseSPEL program and Variable definitions. The corresponding pulse sequence:
    p0−d1−p0−d3−p0−d1−echo.
    In which d1 is fixed and d3 is incremented by a defined step called d30. Contrary to the T_m_ experiment, the 3pESEEM is a single point experiment and we are only monitoring the height of the echo while incrementing the d3.
    The delay d3 was incremented from 400 ns in 8 ns steps and the number of data points was set to 760.
  b. Run a field sweep as explained above (step 81) and set the magnetic center to the value corresponding to the maximum intensity at which the 3pESEEM experiment will be recorded. In Experiment submenu, select 3P ESE setup and in Phase cycle select 4-steps. This setup experiment helps to optimize the stimulated echo by adjusting, the phase, the power attenuation, and the video gain.
  c. Once the echo is optimized, set the variable values for the 3pESEEM experiment as listed below (Table 5):

**Table 5:** Variable values and definitions used for the 3pESEEM experiment

Variable	Value	Definition
p0	16 ns	Pi/2 pulse length.
d1	142 or 454 ns	First pulse delay.
d3	400 ns	Second pulse delay.
d0	410 ns	Acquisition trigger.
Pg	40 ns	Integration gate.
d30	8 ns	Displacement.
SRT	3000 × 1.02 μs	Shot Repetition time.
H	50	Shot Per Point.
N	1	Number of scans.
  d. Press the Run button.
  e. Save the spectrum.
    ***Note:*** The d1 value is inversely proportional to the Larmor frequency of the nucleus under investigation and therefore it is magnetic field dependent, and one needs to adjust it accordingly to the value of the magnetic field at which the 3pESEEM is recorded.
    ***Note:*** Anytime you adjust d1, run the 3pESEEM experiment in Specjet in a similar way as mentioned in step 84e and check that the signal is not clipped. This is due to the fact that the stimulated echo is modulated as the delay d3 is incremented and in some cases you can mistakenly adjust the video gain to an echo that its amplitude corresponds to the lowest point of the modulation. Therefore, you can get a clipped signal by the video gain when signal moves to the highest point in the modulation. This step helps to adjust properly the video gain.

86. For solvent accessibility determination, as we are only interested in deuterium’s contribution, record all 3pESEEM data with a d1 that corresponds to the proton blind spot.
87. Process the data initially with the Bruker Xepr program.
  a. Load the time trace and phase the imaginary part of the signal until you get almost a flat signal.
  b. Extract the real part of the dataset and apply a polynomial baseline correction from the correction task button.
  c. Subtract the baseline and transfer the result to the primary dataset for further processing.
  d. Click the window Function task button and select the Hamming button in the Window Function task bar. Click apply. This will result in a time trace with an amplitude that varies smoothly and gradually toward zero at the end of the trace. This in turn significantly reduces the artefacts that might occur when performing FFT.
  e. Finally, zero fill the data by clicking in the Transformations submenu, of the Processing menu, Zero filling and set in New Length 1024 and click Fill.
  f. Select FFT Real command in the Processing menu and the result will be displayed in the Primary dataset.
  g. Select the Absolute button in the Complex submenu of the Processing menu to get the magnitude spectrum.
88. Use one of the two major approaches to determine the solvent accessibility.
  ***Note:*** The first one is to estimate the solvent accessibility directly from the intensities of the deuterium peaks in the magnitude ESEEM spectra for the different mutants. The second approach is based on a model developed by Jeschke and co-workers which is based on deuterium modulation depth to estimate the solvent accessibility (Volkov et al., 2009). Both methods can provide an estimate of the distance and number of deuterium nuclear spins in the proximity of the nitroxide electron spin and have shown good agreement between their respective results. Troubleshoot 5.
  a. For the first approach, measure the deuterium peak intensity in the magnitude ESEEM spectra obtained for each mutant (after step 87g), to get a direct estimation of its associated solvent accessibility.
  b. For the second approach, fit the time trace obtained after the background correction (spectra obtained after step 87c) by using a damped harmonic oscillation function:
    $$F\left(t\right)=k_{D}\;\cos\left(2\pi \nu_{D}t+\text{Φ}\right)\text{exp}\left(\frac{-t^{2}}{\tau_{0}^{2}}\right)$$
    Where *k*_*D*_ is the deuterium modulation depth, *τ*_0_ is a damping constant, and *φ* the phase, and they are free parameters. The *υ*_*D*_ is the Larmor frequency of the deuterium and is kept fixed, t is the time axis of the time trace.
    The fit can be performed by the curve fitting toolbox in MATLAB.
  c. Use the *k*_*D*_ values determined by the fit and then calculate the solvent accessibility parameter $\text{Π}\left(D_{2}O\right)$ using the formula:
    $$\text{Π}\left(D_{2}O\right)=\frac{2k_{D}}{\left[1-\text{cos}(2\pi \nu_{D}d_{1})\right]}\times {\left(\frac{\nu_{D}}{2\;MHz}\right)}^{2}$$

d1 is the first delay used in the individual 3pESEEM experiment, the deuterium Larmor frequency. *υ*_*D*_ is normalized to 2 MHz.

## Expected outcomes

**Figure 1:**
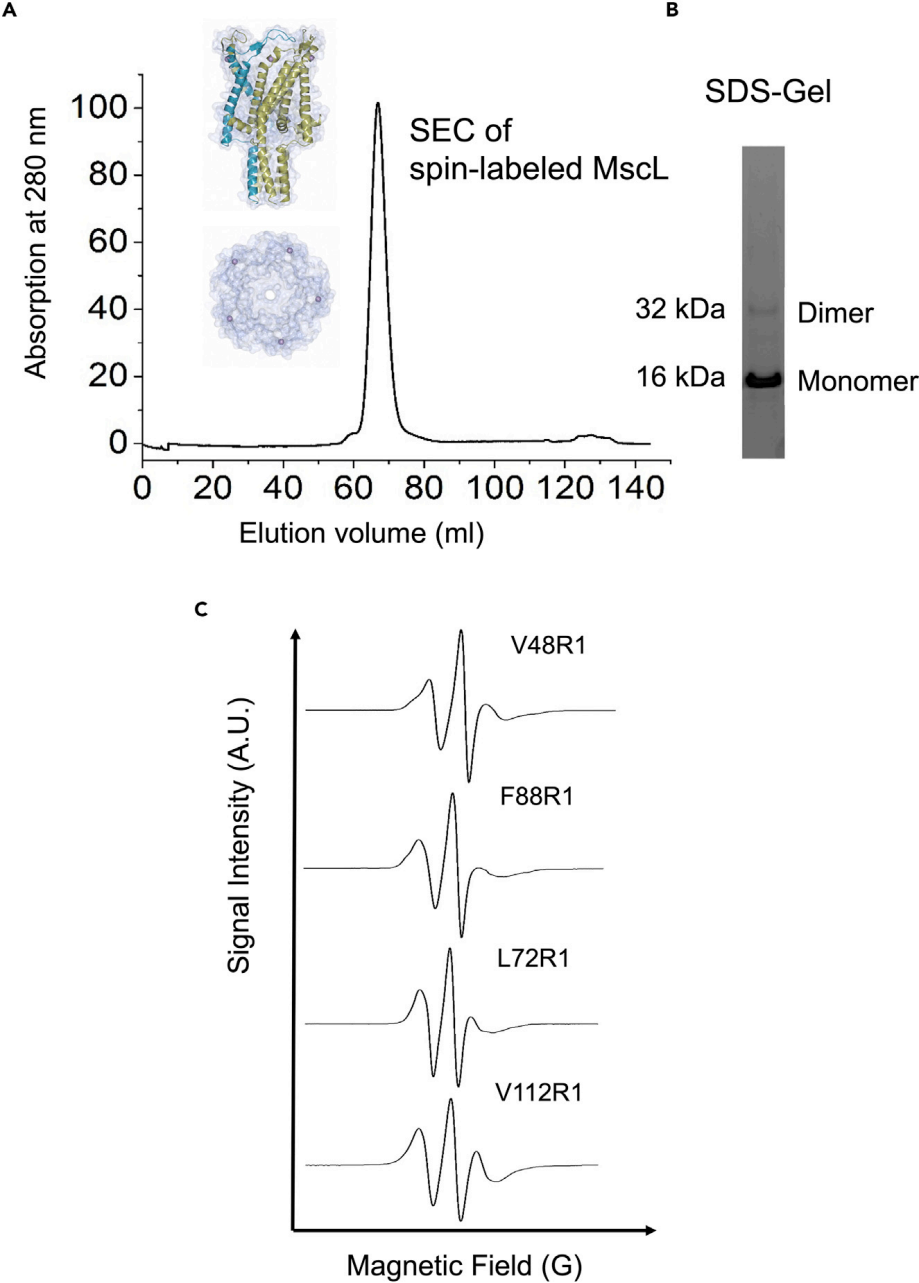
Protein preparation and spin-labeling of MscL mutants (A) A size exclusion chromatography (SEC) profile of spin labeled TbMscL mutant (L89W) representative of multimeric TbMscL on a Superdex 200 16/600 column. Purified mutants should be monodisperse and in a pentameric state in DDM solution. (B) Representative SDS gel analysis of different TbMscL mutants (SEC profile peaks). Two distinct bands should be present for each mutant, which correspond to the monomeric and dimeric form(s) of the protein under SDS denaturing conditions. (C) Representative 18°C–25°C temperature cwEPR spectra of a single cysteine MTSSL-modified (R1) TbMscL mutants (V48R1, L72R1, F88R1, and V112R1).

The expected yield for the different MscL mutants will vary as the location of the mutation will affect protein expression and stability. However, for a 4 L growth of MscL we would expect the cell pellet following protein expression to weigh in the region of 8–11 g, the membrane pellet extracted from these cells to be in the region of 2–3 g, and a protein yield in the region of 5–7 mg. The wild type and mutant proteins should all elute around an elution volume of 12 mL on a Superdex 200 Increase 10/300 or 65 mL on a 16/600 SEC column (Cytiva). The peak on the SEC profile should indicate a homogeneous protein population with a monodisperse pentameric state (Figure 1A). SDS-PAGE analysis can be used in addition to SEC to check sample purity. Two principal bands are expected around 15 and 30 kDa, representing the monomeric and dimeric form of the protein (Figure 1B). If the sample is to be used for EPR spectroscopy, then labeling needs to be checked. Following measurements in the cwEPR spectrometer, a spectrum of three peaks will indicate the presence of bound spin label (Figure 1C). The signal intensity of the central peak should be above 20 A.U. on the nitroxide cwEPR spectrum (Bruker Magnettech ESR5000).

**Figure 2:**
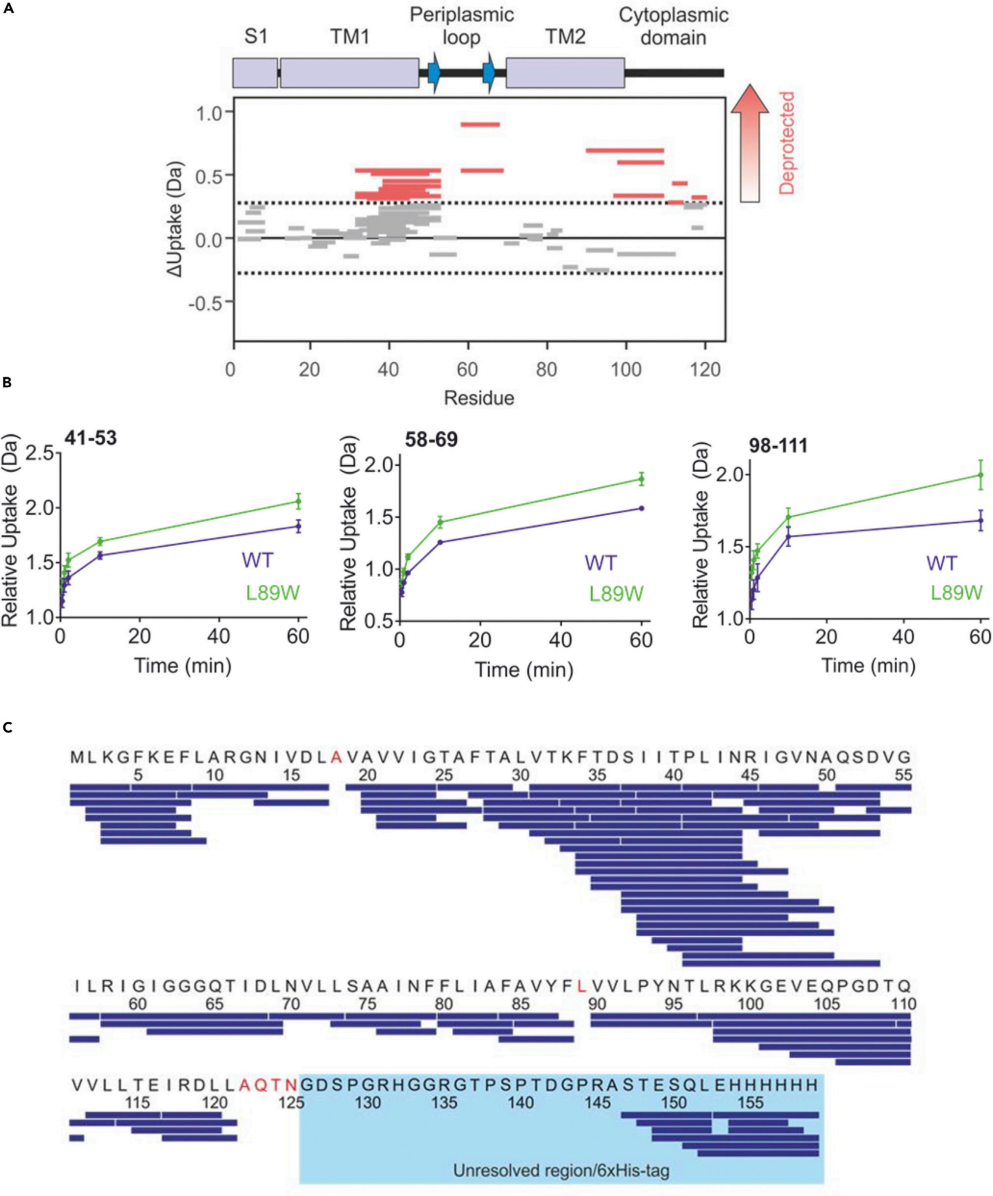
Example HDX-MS data comparing WT MscL with L89W MscL Figure taken from (Wang et al., 2022) showing example precursor HDX-MS data for the protein TbMscL, comparing WT protein with the L89W mutant. (A) A Wood’s plots showing the summed differences in deuterium uptake in MscL, comparing L89W with WT MscL. These Wood’s plots were generated using Deuteros (Lau et al., 2019). Peptides colored in red are deprotected from exchange in L89W MscL in comparison to WT MscL. No peptides were significantly protected from exchange in L89W MscL. Peptides with no significant difference between conditions, determined using a 99% confidence interval (dotted line), are shown in gray. (B) Representative deuterium uptake plots for MscL WT (blue) L89W (green). Residue numbers are indicated in the top left-hand corner. Deuterium uptake plots are shown as mean ± standard deviation of three replicate measurements. Note that the extent of deuterium incorporation increases with incubation time. For the three representative peptides shown the extent of deuterium uptake at all time points is higher in L89W TbMscL compared to WT TbMscL. This demonstrates these regions are deprotected from exchange in the variant studied. (C) A map displaying peptides from MscL detected in the HDX-MS experiment (blue bars). Residues in red are not covered by any of the detected peptides, which totals six residues. The region highlighted in light blue corresponds to the region of the protein that is not resolved in the x-ray structure (2OAR) and the 6×His-tag. Note that we do not detect peptides for most of this unresolved region.

HDX-MS mapping of the MscS protein should generate peptides spanning the majority of the protein (Figure 2A), in our experiments with MscL we achieved 95% peptide coverage of the published MscL structure (2OAR) and 85% peptide coverage of our entire construct. Differential HDX-MS of our two states (WT closed and L89W subconducting) allowed us to identify peptides/regions that undergo significant conformational changes between states. The mass spectra should show an increase in deuterium uptake with incubation over time. Deuterium uptake plots should demonstrate increases in deuteration in the L89W protein vs the wildtype protein for some of the regions of MscL undergoing significant conformational changes. Here we show three deuterium uptake plots (Figure 2B), one from each of the regions that are spanned by peptides with significant deprotection from exchange in L89W compared to WT TbMscL. These three regions comprise residues 37–53 (periplasmic loop), 58–69 (top of TM2), and 97–111 (bottom of TM2, cytoplasmic loop, and top of the cytoplasmic helical bundle). We used this information to direct the selection of single residues for solvent (Deuterium) accessibility measurements via ESEEM spectroscopy. No protection was found for any regions of the protein. The cumulative difference for all the deuterium uptake plots is then plotted for the protein sequence in the form of a Wood’s plot, allowing visualization of the structural dynamics for the entire protein (Figures 2A and 4).

**Figure 3:**
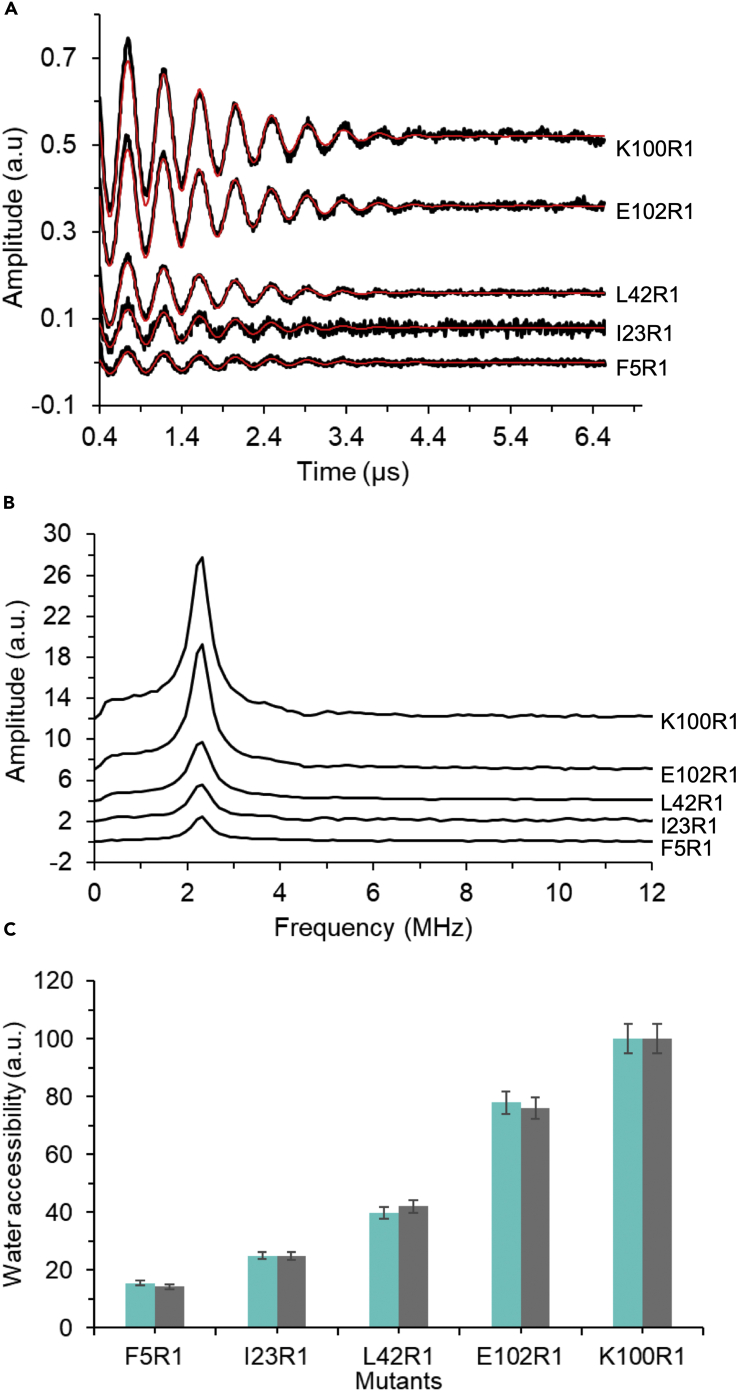
Comparison of deuterium (solvent) accessibility obtained from 3pESEEM time-domain and frequency spectra Figure taken from (Wang et al., 2022). (A) Background-corrected time-domain 3pESEEM raw spectra (black traces) with fitting (red) of representative in solvent exposure levels of spin-labeled mutants across the different MscL domains. F5R1 is found on the S1, I23R1 and L42R1 on TM1, and K100R1 and E102R1 are at the interface between TM2 and the cytoplasmic helical bundle. (B) Frequency domain spectra of 3pESEEM data of F5R1, I23R1, L42R1, K100R1, and E102R1. (C) Column bar charts representing solvent accessibility parameters obtained by two different analysis method approaches. For each sample, the cyan bars correspond to the solvent accessibility derived from the deuterium amplitudes in frequency domain 3pESEEM spectra and normalized to the highest accessibility corresponding to 100%. The grey bars correspond to the solvent accessibility determined from the fitting model to the time domain 3pESEEM spectra and normalized to the highest accessibility corresponding to 100%.

**Figure 4:**
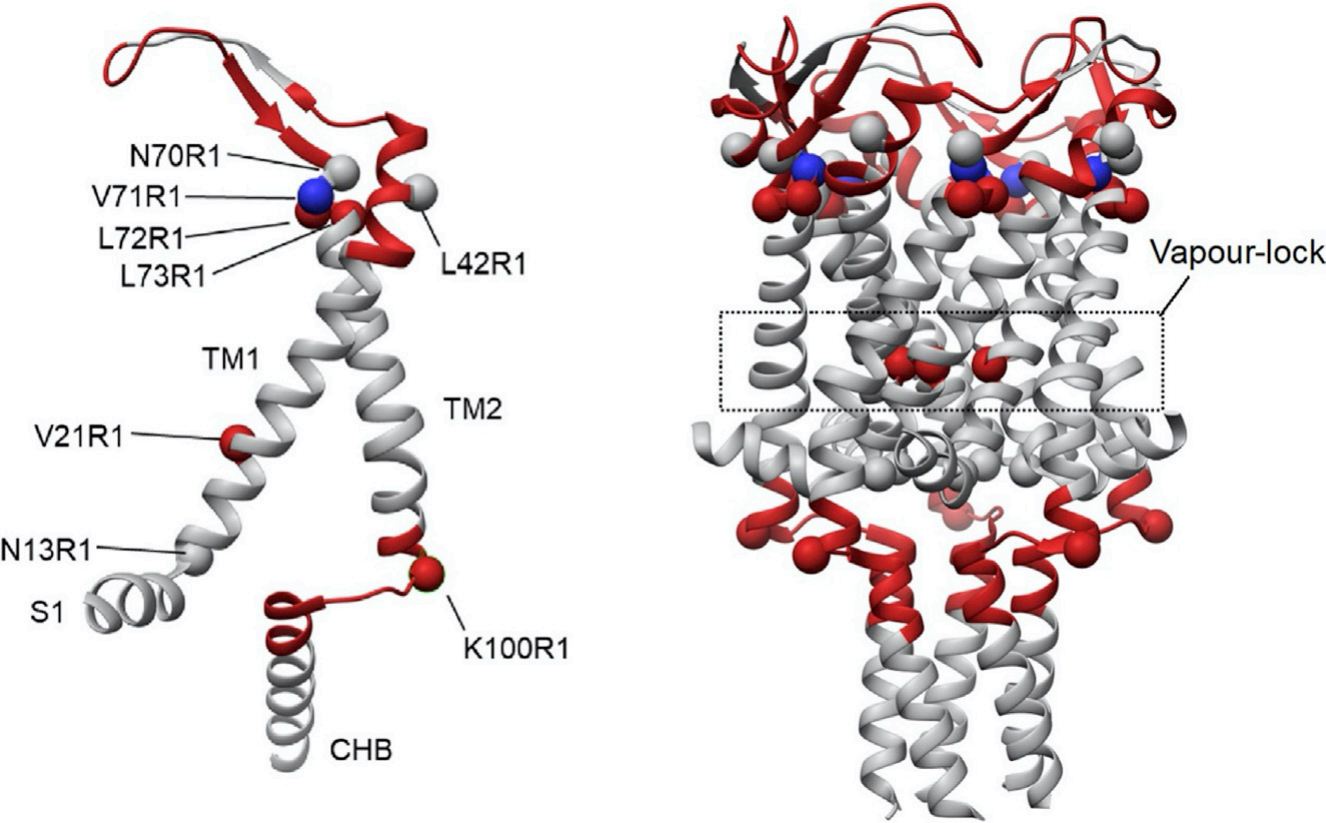
Differences in solvent accessibility for TbMscL (PDB: 2OAR) following L89W modification from HDX-MS and ESEEM spectroscopy Figure taken from (Wang et al., 2022). Spin-labeled mutation sites used for 3pESEEM accessibility measurements are represented by spheres, and peptides that demonstrate a change in accessibility in HDX-MS following the L89W modification are represented as highlighted helices. Red regions or spheres highlight areas that are deprotected, while blue spheres and regions show areas that are protected following the L89W modification. The dynamic regions identified through HDX-MS informed the selection of sites to be labeled for ESEEM spectroscopy. There was no significant difference in the solvent accessibility of N13R1, L42R1, and N70R1 compared to their L89W double-mutant counterpart. Solvent accessibility increased for V21R1 and L72R1 and decreased for L71R1 and K100R1 following the L89W modification.

Solvent accessibility of spin-labeled residues can be determined from the deuterium 3pESEEM (where a τ that corresponds to the proton blind spot is used). We selected single residues to label and probe using ESEEM spectroscopy within regions that were identified as dynamic by HDX-MS, except for V21R1 which is a key residue that forms the vapor lock. We primarily used the obtained time-domains (Figure 3A) traces which are background-corrected, apodized with a hamming window and zero-filled before Fourier transformation. We then use a model developed by Jeschke and Volkov et al. (2009) based on the deuterium modulation depth. The obtained nuclear modulation function is fitted for each mutant using a damped harmonic oscillation function which is then used to estimate solvent accessibility (Volkov et al., 2009). An alternative is to Fourier transform the nuclear modulation function and then the solvent accessibility can be determined directly from the intensities of the deuterium peaks that are present in frequency domain spectra (Figure 3B). Both methodologies should be in good agreement, and we found that solvent accessibility determined by both methods was within the error for TbMscL (Figure 3C). In general, spin-labeled residues within the TM domain showed significantly lower solvent accessibility for MscL, in comparison to selected residues in the periplasmic and cytoplasmic regions. When a subset of these residues (N70R1, V71R1, L72R1, L73R1) on the top of TM2 were paired with the L89W mutation that stabilizes the subconducting state, we were able to demonstrate that the TM2 helix rotates upon channel activation (Figure 4). The spin-labeled V21R1 at the channel vapor-lock showed increase solvent accessibility when paired with L89W, consistent with pore hydration.

## Limitations

Many membrane proteins rely on specific lipids and/or natural substrates for their structural and functional integrity (Laganowsky et al., 2014; Pliotas et al., 2017). Overexpression of membrane proteins that require these low abundance lipids may result in protein that is unstable or in structural conformations that are not physiologically relevant. It also might not be possible to solubilize your membrane protein of interest in a detergent that is amenable to the downstream techniques such as HDX-MS.

A potential limitation of the HDX-MS methodology is that deuterium uptake indirectly reports on conformational dynamics of the backbone of proteins as HDX-MS experiments modify all amide hydrogens across the entire protein as a function of solvent accessibility. Relating these changes to the structural dynamics of a protein is sometimes difficult. Therefore, we investigate structural dynamics within simple comparisons between two states with global structural transitions. In our case, between a closed and expanded (subconducting) channel. Another potential limitation is that areas of specific interest might not digest well in the HDX-MS experiment, disrupting the interpretation of structural dynamics. HDX-MS of very large proteins will significantly increase the length of time for data analysis as a result of the increased number of peptides. Furthermore, solvent accessibility measurements are largely limited to peptide-level resolution in HDX-MS. At the top of TM2, HDX-MS measurements show deuterium uptake for peptides in this region consistent with deprotection in expanded state (L89W). However, single-residue solvent accessibility measurements by ESEEM of four consecutive residues (N70R1, V71R1, L72R1, L73R1) within this peptide showed differential changes in solvent accessibility, with two residues showing deprotection, one residue showing protection, and one residue unchanged. This is consistent with rotation of the TM2 helix and demonstrates a limitation in the interpretability of peptide-level solvent accessibility (Figure 4). Advancements in MS fragmentation technologies, such as electron transfer dissociation, and data deconvolution algorithms are allowing residue-level uptake to be analyzed using HDX-MS. However, this is not trivial, so we utilize ESEEM spectroscopy in the investigation of residue-level solvent accessibility and used HDX-MS as a precursor method for spin labeling strategies and high resolution EPR applications.

One limitation of the use of EPR spectroscopy in the investigation of membrane protein structural dynamics is the requirement to covalently label the protein with a spin-label. Covalent labels can sometimes affect protein structure and stability, although the small size (equivalent to a histidine and much smaller than the fluorophores used in FRET) of an MTSSL spin-label reduces these chances. For our ion channels, we use electrophysiology to ensure any labels are not affecting protein function and relevant functional assays can be used for other classes of proteins. Not all proteins are amenable to EPR spectroscopy *via* site directed spin labeling (SDSL). For the labeling of a specific cysteine residue, native cysteines must be mutated to a serine or an alanine to avoid non-specific labeling, unless they are either buried or form disulfide bridges. Proteins that have a great number of native cysteines that are not involved in disulfide bonding or key functional and conserved cysteines that cannot be mutated are unsuitable for SDSL. However, there are some alternatives such as copper labeling of histidines (Ackermann et al., 2021; Wort et al., 2019; Singewald et al., 2020). The process of SDSL in order to measure solvent accessibility means the technique is more laborious than HDX-MS, but it comes with the benefit of higher resolution. Additionally, EPR does not require a reference sample and the protein samples can be directly frozen under conditions they are already in, without any limitation. Finally, the technique is only semi-quantitative as the most and least exposed residues must be used as a reference to normalize the solvent accessibility of other residues in an experiment.

## Troubleshooting

### Problem 1

Membrane protein precipitation and/or dissociation.

### Potential solution

Detergents used for membrane protein extraction and solubilization are a vital part of the purification protocols. If detergents after the solubilization stage are below or near the critical micellar concentration (CMC), this will likely lead to extensive protein aggregation. Therefore, in such cases an increase to ∼4 CMCs is recommended to check whether this solves the problem. Alternatively, in the case of high symmetric oligomers such as ion channels, we have observed an excess of detergent during and after solubilization may also cause a dissociation of the channel into non biologically relevant low oligomeric state subunits, e.g., monomers and dimers. This is mainly caused due to extensive over delipidation by the detergent which removes lipids that are essential for the membrane protein oligomeric assembly, as is the case for mechanosensitive ion channels (Edwards et al., 2012). In such cases, a gradual reduction of detergent concentration and testing by SEC could identify optimal detergent concentrations that will not dissociate the oligomeric assemblies. Therefore, the detergent type and concentration balance are essential for membrane protein stability and should be optimized through the testing of multiple conditions prior to EPR and/or HDX experiments.

### Problem 2

Low signal on the cwEPR (and subsequently pulsed EPR) spectrometer generated by the spin-labeled protein.

### Potential solution

There is likely to be insufficient labeling of the protein. In such cases increase the concentration of MTSSL, so that it is at least 10 times the expected protein concentration. If this does not result in increased signal, then the low labeling efficiency may be a result of the chosen site (all MscL single Cys mutants used in this study should label sufficiently). Efficiency is dependent on the chosen site and sites that have low accessibility as a result of being buried or obstructed by lipids and/or detergent may not be possible to spin label. Existing structural models can help with the selection of residues for spin-labeling but be aware that these models may not have resolved bound lipids and/or detergent or be in irrelevant conformations.

### Problem 3

Low peptide coverage, poor peptide redundancy, or low peptide signal.

### Potential solution

The mapping likely needs further optimization. The components of the quench buffer can be adjusted such as the salt concentration, addition of a reducing agent, or denaturant (i.e., either urea or guanidinium hydrochloride). Additional proteases can be incorporated with the original protease or alternative proteases can be used.

### Problem 4

As mentioned in step 82, we stated that echo decay and 2pESSEM experiments both have similar setup in terms of number of pulses and that in both experiments the last pulse is incremented with a desired step. Therefore, one should expect to get a similar outcome. However, there is a factor of two to be considered in the data analysis. When performing the echo decay experiment (Tm) we measure the dephasing process occurring between the first pulse and the time of the echo forming (2 × d1, see pulse sequence in step 82) while the 2pESEEM experiment is the result of measuring the event occurring after the last pulse.

### Potential solution

To avoid this, we strongly recommend using the appropriate PulseSPEL programs associated with each type of experiment, although this means that you do have to run a similar experiment twice. In the associated PulseSPEL programs the step is incremented twice in the case of Tm and only once in the case of 2pESEEM. However, you can still determine the Tm and 2pESEEM frequencies from a single experiment but you need to either multiply or divide the X-axes of the result by a factor of two.

### Problem 5

For the solvent accessibility determination by ESEEM, we described two approaches that can be used (see step 88). In the case of using the deuterium intensity, this approach assumes that the concentration of the non-solvent protons in contact with the spin label is invariable regardless of the position of the spin-label on the protein. This assumption is not always true, particularly in the case of membrane proteins, and this might introduce uncertainties.

### Potential solution

We recommend either to use both methods or the approach based on the modulation depth as this parameter is a direct measure of the distance and the local concentration of the deuterium nuclei.

## Resource availability

### Lead contact

Further information and requests for resources and reagents should be directed to and will be fulfilled by the lead contact, Dr Christos Pliotas, c.pliotas@leeds.ac.uk.

### Materials availability

All unique reagents generated in this study are available from the lead contact upon reasonable request.

## Data Availability

HDX mass spectrometry. HDX-MS data have been deposited to the ProteomeXchange Consortium via the PRIDE partner repository with the dataset identifier ProteomeXchange: PXD021983. 3pESEEM. Data are available within the following link: http://archive.researchdata.leeds.ac.uk/777/ and via the https://doi.org/10.5518/914. This paper does not report original code. Any additional information required to reanalyze the data reported is available from the lead contact upon request.
